# Animal Models for Studying Developmental Origins of Cardiovascular–Kidney–Metabolic Syndrome

**DOI:** 10.3390/biomedicines13020452

**Published:** 2025-02-12

**Authors:** You-Lin Tain, Ying-Jui Lin, Chien-Ning Hsu

**Affiliations:** 1 Division of Pediatric Nephrology, Kaohsiung Chang Gung Memorial Hospital, Kaohsiung 833, Taiwan; tainyl@cgmh.org.tw; 2 College of Medicine, Chang Gung University, Taoyuan 333, Taiwan; 3 Department of Pediatrics, Kaohsiung Municipal Ta-Tung Hospital, Kaohsiung 801, Taiwan; 4 Division of Critical Care, Department of Pediatrics, Kaohsiung Chang Gung Memorial Hospital and Chang Gung University College of Medicine, Kaohsiung 833, Taiwan; rayray@cgmh.org.tw; 5 Division of Cardiology, Department of Pediatrics, Kaohsiung Chang Gung Memorial Hospital and Chang Gung University College of Medicine, Kaohsiung 833, Taiwan; 6 Department of Respiratory Therapy, Kaohsiung Chang Gung Memorial Hospital and Chang Gung University College of Medicine, Kaohsiung 833, Taiwan; 7 Department of Early Childhood Care and Education, Cheng Shiu University, Kaohsiung 833, Taiwan; 8 Department of Pharmacy, Kaohsiung Chang Gung Memorial Hospital, Kaohsiung 833, Taiwan; 9 School of Pharmacy, Kaohsiung Medical University, Kaohsiung 807, Taiwan; 10 Depatrtment of Pharmacy, Kaohsiung Municipal Ta-Tung Hospital, Kaohsiung 801, Taiwan

**Keywords:** animal model, developmental origins of health and disease (DOHaD), oxidative stress, microbiota, cardiovascular disease, metabolic syndrome, renin–angiotensin system, kidney disease, nitric oxide, epigenetic regulation

## Abstract

Cardiovascular–kidney–metabolic syndrome (CKMS) has become a significant global health challenge. Since CKMS often originates early in life, as outlined by the developmental origins of health and disease (DOHaD) concept, prevention is a more effective strategy than treatment. Various animal models, classified by environmental exposures or mechanisms, are used to explore the developmental origins of CKMS. However, no single model can fully replicate all aspects of CKMS or its clinical stages, limiting the advancement of preventive and therapeutic strategies. This review aims to assist researchers by comparing the strengths and limitations of common animal models used in CKMS programming studies and highlighting key considerations for selecting suitable models.

## 1. Introduction

Cardiovascular disease (CVD) is responsible for approximately one-third of all deaths worldwide [1]. In 2013, the World Health Organization set a global target to reduce premature mortality from non-communicable diseases, including CVD, by 25% [2]. While reductions in many leading causes of death contributed to increased life expectancy across most regions between 1990 and 2021, these gains were significantly affected by the setbacks of the COVID-19 pandemic [3]. Given that COVID-19 may influence CVD and cardiovascular risk [4], CVD remains the leading cause of global deaths, underscoring the need for further reductions.

CVD frequently coexists with obesity, kidney disease, and type 2 diabetes [5]. For example, cardiorenal syndrome is a complex, multi-organ condition involving dysfunction of the heart, kidneys, and vascular system [6]. Moreover, individuals with chronic kidney disease (CKD) are at a significantly higher risk of developing CVD [7]. Similarly, metabolic syndrome—a cluster of conditions—substantially increases susceptibility to CVD [8]. The concept of cardiometabolic syndrome has emerged, combining features of metabolic syndrome with additional cardiovascular risk factors, with multiple conditions significantly amplifying health and mortality risks, particularly from CVD [9]. Recognizing this intricate interplay, the American Heart Association (AHA) has introduced the term cardiovascular–kidney–metabolic syndrome (CKMS) to define these adverse interconnections [10].

CKMS is classified into four stages (1–4), representing varying degrees of severity and progression within its complex spectrum [11]. The main components develop at different stages, driving the progression and impact of the syndrome. Approximately 90% of adults in the United States are estimated to be affected by CKMS [12]. By primarily increasing cardiovascular risks, CKMS contributes significantly to the global disease burden. Holistic management that addresses the entire syndrome rather than individual conditions is recommended for optimal care [12], though comprehensive guidelines are yet to be established. Early prevention and recognition of the interconnected organ systems in CKMS are crucial for mitigating the global burden of CVD.

Growing evidence suggests that adverse conditions during pregnancy and early infancy increase susceptibility to adult CKMS [13,14,15,16]. The developmental origins of health and disease (DOHaD) theory suggests that a developing fetus adapts to environmental cues, potentially increasing the risk of chronic diseases in adulthood [17]. However, these disease-related programming processes could potentially be reversed by shifting therapeutic interventions from adulthood to the perinatal period, a concept referred to as reprogramming [18]. Given that CKMS may originate early in life, preventing rather than merely treating CKMS within the DOHaD framework presents a promising new strategy.

Several hypotheses, including the thrifty phenotype [19], predictive adaptive responses [20], lifespan development theory [21], and maternal capital [22], have been proposed to explain links between early life insults and adult diseases. However, these theories do not outline specific mechanisms or preventive strategies. Animal models have been crucial in establishing the biological plausibility of these associations and providing proof of causality. Emerging evidence highlights their value in understanding developmental programming and creating therapies for DOHaD-related diseases [23,24,25,26]. Both small (e.g., rats, mice) and large (e.g., pigs, sheep) animal models are utilized, each offering unique advantages.

This review offers a comprehensive overview of the critical contributions of animal models to the field of DOHaD research, with a particular focus on their role in elucidating the developmental origins of CKMS. Insights gained from various animal models have helped to establish key mechanisms involved in the developmental origins of CKMS. Integrating evidence from diverse animal models is essential not only for identifying novel therapeutic targets but also for informing the development of innovative reprogramming strategies to reverse or mitigate adverse developmental programming. These efforts are pivotal in addressing the escalating global burden of CVD and related disorders.

## 2. Material and Methods

We conducted a comprehensive literature review by identifying relevant studies published in English between January 2000 and December 2024. The review includes animal research and clinical studies sourced from scientific databases, such as MEDLINE, Embase, and the Cochrane Library, with an emphasis on full-text articles in English. The search employed key terms related to animal models, DOHaD, and CKMS. The employed search terms included “animal model”, “metabolic syndrome”, “obesity”, “chronic kidney disease”, “cardiovascular disease”, “hypertension”, “hyperlipidemia”, “dyslipidemia”, “insulin resistance”, “hyperglycemia”, “diabetes”, “liver steatosis”, “atherosclerosis”, “heart failure”, “developmental programming”, “DOHaD”, “offspring”, “progeny”, “mother”, “oxidative stress”, “reprogramming”, “gut microbiota”, “pregnancy”, and “lactation”. Additionally, we thoroughly reviewed the reference lists of articles to identify any other relevant sources for this review.

## 3. Choice of Animal Models

A diverse array of animal models has been established to confirm the associations found in human observational studies, allowing these findings to be reproduced under controlled experimental conditions in DOHaD research [23]. These models can be categorized in several ways.

First, animal models in DOHaD research can be classified based on the types of environmental exposures in early life. For instance, caloric restriction in animals can mimic the starvation conditions seen in human populations during famine [27]. Second, these models can also be categorized based on the molecular mechanisms they aim to test. Numerous animal models have been developed to investigate these proposed mechanisms, as different early-life environmental cues often lead to similar outcomes concerning specific components of CKMS in adult offspring. This suggests that common mechanisms may underlie the adverse processes involved in developmental programming.

Finally, animal models used in DOHaD research encompass a range of small- and large-animal species, each with distinct strengths and limitations [8]. Non-human primates have long been considered the gold standard due to their high genetic and biological similarity to humans. However, the most commonly used species in DOHaD research are rodents, specifically rats and mice, which offer cost-effective options with short life cycles and ease of handling. Additionally, mice are especially useful for genetic modification. Other species, such as pigs, sheep, and rabbits, have also been utilized to study developmental programming and offspring outcomes [27]. Pigs are ideal for studying early fertilization and development, while sheep, with their long gestation period, have fetal sizes and developmental rates similar to humans [28]. Rabbits are valuable for research due to their placental structure and lipid metabolism, which are similar to humans [29]. Cattle, with similar gestation lengths to humans and typically singleton pregnancies, are also utilized [28]. Selecting an appropriate animal model requires considering factors like genetic background, anatomy, physiology, gestation length, litter size, and clinical relevance.

A variety of animal models exposed to different environmental cues have been developed to examine specific aspects of CKMS, including CVD [30], hypertension [24,31], kidney disease [32,33], and metabolic syndrome [34], as reviewed in previous studies. Given the multi-organ dysfunction characteristic of CKMS and its distinct components, only animal models that successfully replicate at least two components of CKMS in adult offspring are summarized in Table 1.

Various suboptimal environmental conditions during gestation and breastfeeding have been linked to different aspects of CKMS in adult offspring. These include maternal nutritional imbalances, maternal conditions, drug use, and chemical exposure. Each category is discussed in detail below.

### 3.1. Maternal Nutritional Imbalance

In DOHaD research, animal model studies on nutritional programming have been conducted for decades [23,110]. Both macronutrients (e.g., carbohydrates, fats, and proteins) and micronutrients (fatty acids, amino acids, vitamins, and minerals) have been extensively investigated. It is well established that excessive or insufficient intake of specific nutrients can trigger aspects of CKMS of developmental origins in animal models, as summarized in Table 1. Among the most frequently employed models are those involving nutritional manipulations during gestation and breastfeeding, including caloric restriction, protein restriction, overnutrition (achieved through litter size reduction), high-fructose diets, and high-fat diets.

Caloric restriction entails a reduction in overall energy and nutrient intake, mimicking conditions of human famine. Studies have shown that caloric restriction ranging from 30% to 70% in pregnant rats can induce hypertension in their adult offspring, with more severe restrictions leading to earlier onset of hypertension [111]. Similar outcomes have been observed in other species, such as sheep [38] and pigs [39], where caloric restriction induces various characteristics of CKMS in adult offspring.

The protein restriction model has also been extensively utilized to investigate the mechanisms underlying nutritional programming [112]. As with caloric restriction, protein restriction during gestation and breastfeeding predisposes adult offspring to obesity, insulin resistance, diabetes, hypertension, CVD, and kidney disease—key components of CKMS [41,42,43,44,45].

Deficiencies in micronutrients such as zinc [113], iron [114], vitamin D [115], sodium [116], and calcium [117] during pregnancy have also been linked to offspring hypertension. However, it remains largely unclear whether these maternal micronutrient deficiencies contribute to the developmental origins of other CKMS components.

On the other hand, maternal overnutrition contributes significantly to offspring CKMS. Litter size reduction during lactation has been used as an experimental method to induce overfeeding, accelerate neonatal growth, and promote early onset of overweight/obesity in rodents [118]. As shown in Table 1, offspring in this model often display obesity, insulin resistance, hypertension, and kidney disease [46,47,48].

With the rising prevalence of fructose-rich diets and their connection to CKMS [119], the impact of excessive maternal fructose consumption on offspring health has become increasingly significant [120]. A maternal high-fructose diet is a widely used rodent model to study CKMS of developmental origins, covering outcomes such as obesity, insulin resistance, hypertension, and dyslipidemia [49,50,51,52,53,54,55].

Animal models consistently show that high-fat diets contribute to obesity and related diseases [121,122]. Evidence from studies using various animal species indicates that offspring exposed to a maternal high-fat diet exhibit all characteristics of CKMS, including obesity, hypertension, insulin resistance, dyslipidemia, metabolic dysfunction-associated steatotic liver disease (MASLD), and kidney disease [56,57,58,59,60,61,62,63].

Studies using animal models of nutritional programming are invaluable due to the critical role of maternal nutrition in shaping fetal growth and development during pregnancy and breastfeeding. These models enhance our understanding of the timing, optimal dose, and duration of nutritional interventions, thereby informing clinical practice.

### 3.2. Maternal Health Conditions

Maternal health conditions impact fetal programming and increase the risk of developing CKMS in offspring. To investigate these effects, animal models that simulate maternal illnesses and pregnancy complications have been established, with rats being the most commonly used species.

Gestational diabetes mellitus is the most common pregnancy complication, which is linked to maternal health issues and long-term adverse outcomes for offspring [123]. In animal studies, a single dose of streptozotocin (STZ) induces type 1 diabetes in adult rats, whereas neonatal STZ injection (nSTZ) produces type 2 diabetes in adulthood [124]. Both STZ-induced diabetic rats and nSTZ rats have been used as models to demonstrate an association between maternal diabetes and CKMS development in offspring [64,65,66,67,68].

Pregnant women with CKD face higher risks of adverse outcomes for themselves and their offspring [125]. An adenine-induced maternal CKD model is used to study uremia-related complications during pregnancy and their impact on offspring, including the developmental origins of kidney disease and hypertension [69,70]. However, its programming effects on other components of CKMS remain largely unknown.

Another common pregnancy complication, uteroplacental insufficiency, has been linked to various features of CKMS, including dyslipidemia, insulin resistance, hypertension, and kidney disease [71,72,73,74,75]. Similarly, offspring CKMS can be programmed by maternal hypoxia in animal models such as rats [76,77,78], mice [79], and sheep [80]. Research has also identified a link between hypoprolactinemia and the development of metabolic syndrome and impaired cardiometabolic health [126]. Rat models of maternal hypoprolactinemia have been utilized to induce CKMS in offspring, including obesity, insulin resistance, and kidney disease [81,82]. Prenatal exposure to pyrogens such as lipopolysaccharide (LPS) and zymosan has been employed to mimic maternal inflammation. Maternal LPS exposure has been revealed to increase adiposity and disrupt metabolic homeostasis in offspring [83]. Additionally, both maternal inflammation models have demonstrated elevated blood pressure (BP) in adult offspring [84,85].

### 3.3. Drug Use

Numerous medications given to pregnant women can impact fetal kidney development, potentially leading to kidney disease later in life [127]. These medications include aminoglycosides, angiotensin-converting enzyme inhibitors (ACEIs), angiotensin receptor blockers (ARBs), cyclosporine, nonsteroidal anti-inflammatory drugs, dexamethasone, furosemide, Adriamycin, anti-epileptic drugs, and cyclophosphamide. Among these, the use of cyclosporine A [128], dexamethasone [129], and gentamicin [130] during pregnancy has been connected to reduced nephron numbers in animal models. This reduction is associated with an increased risk of kidney disease and hypertension in adulthood. However, the programming effects of these medications on cardiovascular and metabolic outcomes remain unclear and warrant further investigation. Additionally, studies have shown that the use of metformin during lactation induces metabolic programming [131], though its impact on cardiovascular systems and kidney function has not yet been explored. Therefore, the long-term programming effects of these medications on offspring, particularly in the context of CKMS, remain insufficiently studied and require further research.

In utero exposure to synthetic glucocorticoids has been reported to induce obesity, insulin resistance, hypertension, and kidney disease in adult offspring [86,87,88,89,90,91]. Glucocorticoids play a crucial role in regulating the hypothalamic–pituitary–adrenal axis, which governs various organ systems, including the kidneys, liver, and endocrine system. Emerging evidence suggests that glucocorticoid programming involves permanent, organ-specific changes in gene expression, particularly of the glucocorticoid receptor itself [132].

Substance abuse is a major maternal insult, with 6–16% of pregnant women in the U.S. smoking, drinking alcohol, or using illicit drugs during pregnancy [133]. Studies in rat models have shown that maternal nicotine exposure negatively impacts offspring, leading to hyperlipidemia, steatosis, hypertension, and kidney disease [92,93,94,95]. Similarly, maternal ethanol exposure has been associated with insulin resistance, kidney disease, and CVD in adult offspring [96,97,98]. While observational studies have linked illicit drug exposure during pregnancy to adverse late-life phenotypes [134], the effects of maternal illicit drug use on offspring’s cardiovascular, kidney, and metabolic health remain largely unexplored.

### 3.4. Chemical Exposure

Environmental chemicals can act as endocrine-disrupting chemicals, impacting placental formation and fetal development [135]. These chemicals may increase the risk of pregnancy complications and induce cross-generational effects through epigenetic mechanisms.

Numerous epidemiological studies have explored the associations between background exposure to these chemicals and health outcomes in infants and children [136]. As summarized in Table 1, maternal exposure to substances such as 2,3,7,8-tetrachlorodibenzo-p-dioxin (TCDD) [99,100,101], di-n-butyl phthalate (DEHP) [102,103,104,105,106], and bisphenol A (BPA) [107,108,109] has been linked to the developmental origins of CKMS in adult rodent offspring.

Evidence from these studies shows that maternal health conditions significantly affect offspring susceptibility to CKMS, supporting epidemiological findings. However, the relevance of other maternal conditions to the developmental programming of CKMS remains insufficiently explored. It is also noteworthy that most animal models primarily use rats and often focus on specific aspects of CKMS rather than addressing the syndrome as a whole. To advance DOHaD research, future studies should aim to validate these observed effects through long-term follow-ups using diverse animal species. Such efforts are essential to identify the underlying common mechanisms and enhance our understanding of CKMS developmental programming.

## 4. Mechanisms of CKMS Programming

Animal models can be refined and developed based on the mechanisms shared between humans and these models. Mechanisms associated with the DOHaD theory include oxidative stress, epigenetic dysregulation, disrupted nutrient-sensing signaling pathways, aberrations in the renin–angiotensin system (RAS), gut microbiota dysbiosis, inflammation, and sex-specific differences [23,24,30,31,32,33,34,137,138,139,140]. Several of these mechanisms are directly linked to CKMS programming and will be explored in greater detail.

### 4.1. Oxidative Stress

Oxidative stress occurs when an excess of reactive oxygen species (ROS) overwhelms the antioxidant defense system [141]. Its role in the development of various aspects of CKMS has been extensively studied [142,143,144,145]. During pregnancy, the physiological production of ROS is crucial for normal developmental processes. However, compromised pregnancies are often associated with heightened oxidative stress [146].

Numerous oxidative stress mechanisms have been implicated in CKMS programming [147], including the upregulation of ROS-generating enzymes, increased ROS production, reduced antioxidant availability, enhanced oxidative damage, and disruptions in the nitric oxide (NO) pathway. In CKMS of developmental origin, oxidative stress can affect multiple organs, such as the kidneys [35,86], blood vessels [148], and liver [149]. Elevated ROS-producing enzyme levels and increased ROS production have been observed in various CKMS programming models. These include maternal nicotine exposure [148], maternal glucocorticoid administration [149], and ethanol exposure [150]. In the litter-size reduction model, offspring exhibited obesity and insulin resistance accompanied by reduced activity of antioxidant enzymes, such as superoxide dismutase, catalase, and glutathione peroxidase [47].

Several biomarkers of oxidative damage to proteins, lipids, and DNA have been assessed in animal models of CKMS programming. These include 8-hydroxydeoxyguanosine (8-OHdG) [35], F2-isoprostanes [75], malondialdehyde [151], 4-hydroxynonenal [151], and thiobarbituric acid reactive substances [152].

An additional key contributor to oxidative stress in CKMS pathogenesis is the impaired nitric oxide synthase (NOS)/NO pathway [153]. Elevated levels of the endogenous NOS inhibitor asymmetric dimethylarginine (ADMA) reduce NO production, leading to endothelial dysfunction and exacerbating oxidative stress [154]. This dysfunction has been linked to the development of various CKMS components in models of caloric restriction [35], maternal diabetes [67], CKD [70], and BPA exposure [107].

### 4.2. Aberrant RAS

Angiotensinogen (AGT) is converted into angiotensin I (Ang I) and then angiotensin II (Ang II) by renin and ACE [155]. Ang-(1–7), produced from Ang I and Ang II via ACE2 [156], counteracts the classical RAS pathway by acting through the MAS receptor, opposing the ACE, Ang II, and Ang II type 1 receptor (AT1R) axis.

The RAS is critical for regulating physiological processes such as cardiovascular function, kidney health, and metabolic homeostasis. Dysregulation of the RAS has been implicated in conditions such as hypertension, CVD, kidney disorders, and metabolic syndrome [157,158,159]. Additionally, RAS activation, driven by increased AGT production in adipose tissue, contributes to the pathogenesis of obesity-related metabolic and inflammatory disorders [160].

During kidney development, RAS components are highly expressed and play essential roles in guiding the proper formation and physiological function of the renal system [161]. In rat models, all RAS components are detectable in embryonic kidneys from gestational days 12 to 17, with higher levels in fetuses and newborns than adults [162]. In humans, RAS-interfering medications like ACEIs and ARBs are avoided during pregnancy due to their association with renal malformations and ACEI/ARB fetopathy [127].

Currently, ACEIs and ARBs are indicated for the treatment of CKMS [10], as their use has been linked to improved cardiovascular, kidney, and metabolic benefits in high-risk populations [10]. In the context of CKMS programming, only limited data are available regarding RAS-targeted interventions in early life for CKMS prevention.

Early inhibition of the classic RAS axis is proposed to reprogram aberrantly activated RAS and prevent CKMS. In rodents, kidney development is complete by weeks 1–2, and cardiomyocytes rarely reenter the cell cycle after day 9, making the optimal therapeutic window for treatment initiation around 2 weeks postnatally. Therapeutic interventions during this period include administering aliskiren [163], captopril [164], or losartan [165] between 2 and 4 weeks of age. These approaches aim to mitigate adverse programming effects without disrupting kidney development. Additionally, studies have shown that administering an ACE2 activator or angiotensin-(1–7) [ANG-(1–7)] during pregnancy can alleviate hypertension and kidney disease in adult offspring of spontaneously hypertensive rats [166].

These findings highlight the potential value of developing animal models with organ-specific knockouts or knockdowns of specific RAS components. Such models could provide deeper insights into the role of local RAS in the developmental origins of CKMS and the associated cellular mechanisms.

### 4.3. Epigenetic Modifications

Accumulating evidence links CKMS to epigenetic alterations encompassing a complex interplay of DNA methylation, histone posttranslational modifications, and microRNAs (miRNAs) [167,168,169]. The developmental phase spanning pregnancy to lactation is marked by heightened epigenetic modifications [170].

Animal models suggest that DNA methylation mediates the effects of early life exposures on later obesity risk [171]. Maternal protein restriction is linked to DNA hypermethylation in the livers and adipose tissues of rat offspring [172,173]. Hypertension pathogenesis involves up-regulation of ACE and AT1R with hypomethylation of their promoter regions, as decreased DNMT3a binding to the AT1R gene causes DNA demethylation in a maternal protein restriction model [174,175].

A maternal high-fructose diet induces changes in the renal transcriptome and hypertension in adult offspring [163]. Whole-genome RNA sequencing identified 2706, 1255, and 2147 differentially expressed genes (DEGs) in the kidneys of 1-day, 3-week, and 3-month-old rat offspring, respectively [52]. In neonatal kidneys, seven histone modification-related genes (*Brdt, Brwd1*, *Chd2, Dnmt3l, Hdac9, Hdac11,* and *Myst2*) showed significant regulation exclusively in 1-day-old rat offspring [52]. Since these genes were altered during kidney development, they likely contribute to the epigenetic regulation of kidney development.

In a maternal protein restriction rat model, altered expression of miRNAs, including upregulation of miR-125a-3p, miR-142-3p, miR-182, let-7b, and miR-188-5p in cardiac tissue, alongside downregulation of miR-107, miR-181a, miR-181c, miR-184, miR-127, miR-324-5p, miR-383, miR-423-5p, miR-484, and let-7g, suggests their potential role in the development of offspring CVD [176].

Emerging evidence suggests maternal nutritional imbalances impact metabolic programming and epigenetic changes in offspring [177]. Several studies highlight epigenetic modifications as a mechanism behind the altered development of the hypothalamic appetite and energy balance system. This system, especially within the arcuate nucleus (ARC), develops during embryonic and neonatal life [178], making it sensitive to maternal metabolic and nutritional influences. For example, high-fat diet-induced obesity in rat dams leads to hypermethylation of the *Pomc* promoter in the ARC, affecting offspring from birth to adulthood [179]. Additionally, high-fat diet offspring show reduced DNA methylase expression and suppressed ARC development [180]. Poor maternal nutrition can also influence adipogenesis, with high-fat diet offspring showing increased adipogenic differentiation and altered DNA methylation in fat progenitor cells [181]. These epigenetic modifications may predispose offspring to obesity.

Epigenetic processes, which vary throughout life, may act in a developmental organ- specific manner, linking maternal insults to the developmental origins of CKMS [182]. Therefore, research should focus on uncovering the mechanistic pathways underlying CKMS programming. Rather than exclusively developing animal models based on specific epigenetic patterns, it is essential to prioritize understanding the complex mechanisms involved.

### 4.4. Gut Microbiota Dysbiosis

Emerging evidence highlights the gut microbiome’s key role in CKMS components such as CVD, CKD, obesity, and metabolic syndrome [183,184,185]. Both gut microbes and their metabolites, including altered microbial composition, dysregulated short-chain fatty acids, elevated trimethylamine N-oxide levels, and microbiota-derived uremic toxins, are implicated in CKMS [186,187,188].

Neonatal gut colonization begins at birth [189], with the microbiome diversifying and reaching an adult-like composition by around two years, including the lactation period [190]. Maternal factors such as gestational age, delivery mode, health conditions, antibiotics, and environmental influences shape its development [191]. Breastfeeding may serve as a key link between maternal risks and CKMS programming [192].

Altered gut microbiota and metabolites have been shown to program features of CKMS with developmental origins in animal models, including those involving maternal high-fat diets [56], maternal CKD [69], TCDD exposure [100], DEHP exposure [106], and BPA exposure [107,109]. Conversely, interventions like probiotics, prebiotics, and postbiotics have shown promise in managing CKMS [193,194,195]. Since an infant’s microbiota is shaped by the maternal microbiome, understanding the mechanisms linking gut microbiota to cardiovascular–kidney–metabolic interactions could guide the development of targeted microbiota-based interventions to reduce offspring CKMS risk.

## 5. Selection of Appropriate Animal Models

### 5.1. Key Considerations

Considering the complex multisystem interactions in CKMS, developing animal models is critical for clarifying the molecular mechanisms underlying its development and progression. A deeper understanding and careful selection of animal models that reflect the variability and stages of CKMS could guide novel strategies to predict and improve cardiovascular–kidney–metabolic health in the population.

**Figure 1:**
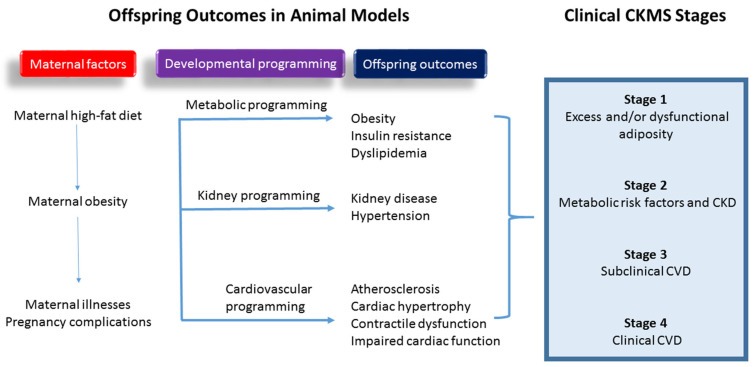
A visual representation of how maternal high-fat diet contributes to the developmental origins of cardiovascular–kidney–metabolic syndrome (CKMS) in offspring and the comparison to clinical CKMS stages.

Despite progress in developing animal models for CKMS of developmental origins, meaningful clinical translation remains a key research priority. While current animal research on CKMS of developmental origins predominantly focuses on rodent models, large animal models are often more physiologically similar to humans and could provide valuable translational insights. There is an unmet need for additional studies using large animal models to enhance the clinical translation of these findings. Additionally, the maternal high-fat diet rodent model replicates all CKMS characteristics, as demonstrated in previous studies [56,57,58,59,60,61,62,63] (Figure 1). However, no study has yet evaluated the stages of CKMS simultaneously to pinpoint the timing of individual phenotype development across the lifespan within a single study.

Key factors in selecting animal models include aligning the timing of organogenesis with humans, ensuring comparable gestation periods and litter sizes, maintaining high methodological quality, and standardizing protocols to produce measurable outcomes that closely reflect human studies.

### 5.2. Timing of Organogenesis

Organ development timing varies across species, influencing the translatability of animal studies. BP regulation involves coordination among the kidney, heart, brain, and other systems, but key stages of organ development differ between humans and animals. For instance, many BP-regulating organs develop prenatally in humans but postnatally in some species [32,196]. Studies on developmental hypertension suggest kidney, cardiovascular, or brain programming as contributors [137,197,198], though few have comprehensively evaluated all BP-regulating systems during specific developmental stages.

In humans, kidney development spans from week 3 to 36 of gestation [199], while in rats, it continues until 1–2 weeks postnatally [200]. Similarly, human brain growth peaks near birth, but in rats, it peaks around postnatal days 7–8 [196]. Adverse conditions during pregnancy and lactation can impair kidney and brain development, leading to hypertension in rodents. Given the interplay between organogenesis and environmental factors, selecting animal models that align closely with human organ development is crucial for accurately assessing early-life insults.

### 5.3. Gestation Period and Litter Size

Rodent models are widely used in DOHaD research due to their shorter pregnancy period and higher offspring yield compared to large animal models. Rats, with a gestation period of about 23 days versus 280 days in humans [201], are particularly common for studying the developmental origins of CKMS (Table 1). However, the short gestation period can limit the resolution of developmental plasticity and the identification of critical vulnerability windows, especially if early-life insults require surgical intervention or repeated procedures.

Rodents typically produce large litters of 8–12 pups, unlike the singleton births common in humans and large animals. This can lead to variations in offspring food intake, maternal care, and growth, necessitating litter size normalization after birth to minimize confounding effects [202]. These factors, along with physiological differences, pose challenges for directly translating rodent findings to human medicine.

Large animal models, such as sheep, offer closer parallels to human development. Sheep have a gestation length of about 150 days, with fetal growth and development rates comparable to humans [203]. Studies using sheep have demonstrated that maternal caloric [38] or protein restriction [45], high-fat diets [63], maternal hypoxia [80], and prenatal glucocorticoid exposure [90] can lead to CKMS in adulthood. However, further research is needed to evaluate how differences in gestation length and litter size influence CKMS development across species.

### 5.4. Methodological Quality and Standardization

This review highlights how various early-life cues influence outcomes, contributing to observed heterogeneity. These results are heavily dependent on the measurement techniques and animal models used, with methodological differences further increasing variability.

Unlike the well-defined criteria for diagnosing overweight and obesity in CKMS staging [10], diagnosing obesity in rats remains inconclusive. It requires evaluating a combination of physical, physiological, and metabolic parameters, as body weight alone is insufficient. Common methods include body composition analysis, fat pad weight measurements, leptin levels, glucose and lipid profiling, and histological analysis of adipose tissues. Notably, hypertension measurements in rodents also vary, with the tail-cuff method commonly used despite its potential to elicit stress-related increases in BP due to sympathetic nerve activity [204]. Although tail cuff measurements are reported to correlate well with telemetry and direct arterial catheter methods [205], the stress factor may partly explain elevated BP findings. Additionally, while non-human primates have been studied for cardiovascular outcomes programmed by maternal adverse exposures [206], they have not been used for developmental hypertension research.

A significant proportion of studies utilize male-only small animal models with small sample sizes, limiting generalizability. Future research should address these issues by implementing randomization, blinding, and appropriate sample size calculations to reduce bias and improve data quality.

Disparities in therapeutic doses, timing, duration, and animal models further complicate comparisons. Standardizing experimental protocols is critical to enhancing comparability across studies. The efficacy of interventions is influenced by their timing and duration relative to organ development, varying by dose and species. Hence, translational research into the metabolism and pharmacokinetics of interventions is necessary to validate their safety and therapeutic potential across species, including humans.

## 6. Conclusions

Despite CKMS and its associated disorders emerging as major public health concerns, specific preventive interventions are still lacking [207]. Various small and large animal models have greatly advanced DOHaD research, linking early-life insults to the risk of CKMS later in life. These models help uncover mechanisms behind CKMS of developmental origins and support the development of early-life interventions for prevention. However, questions remain regarding risk factors affecting offspring CKMS, organ-specific programming processes, and potential pharmacological and non-pharmacological interventions during pregnancy and lactation. While several classes of medications (e.g., sodium-glucose cotransporter-2 inhibitors and glucagon-like peptide-1 receptor agonists), which have demonstrated benefits in slowing the progression of CKD, preventing CVD events, and reducing cardiovascular mortality, have been recommended by the AHA guideline [10], their effects on CKMS of developmental origins have not received as much attention and require further clarification. Preventive strategies should focus on reducing perinatal exposure to harmful factors and promoting healthy lifestyles. Each model targets specific hypotheses and CKMS phenotypes, with none universally superior for all aspects of developmental origins. Further research is crucial to identify early-life insults, understand mechanisms, refine interventions, and select appropriate models, applying the DOHaD approach to mitigate the global burden of CKMS.

## Figures and Tables

**Table 1:** Overview of rodent models utilized for studying developmental origins of CKMS.

Experimental Model	Types of Exposure	Species	Components of CKMS	References
Maternal nutritional imbalance	Caloric restriction	Rats/mice/sheep/pigs	Obesity, insulin resistance, kidney disease, and hypertension	[35,36,37,38,39,40]
	Protein restriction	Rats/mice/sheep	Obesity, insulin resistance, diabetes, hypertension, CVD, and kidney disease	[41,42,43,44,45]
	Litter size reduction	Rats/mice	Obesity, insulin resistance, hypertension, and kidney disease	[46,47,48]
	High-fructose diet	Rats/mice	Obesity, insulin resistance, dyslipidemia, and hypertension	[49,50,51,52,53,54,55]
	High-fat diet	Rats/mice/pigs/sheep	Obesity, insulin resistance, hypertension, dyslipidemia, MASLD, kidney disease, and CVD	[56,57,58,59,60,61,62,63]
Maternal health conditions	Maternal diabetes	Rats/mice	Obesity, insulin resistance, hypertension, dyslipidemia, diabetes, and kidney disease	[64,65,66,67,68]
	Maternal chronic kidney disease	Rats	Hypertension and kidney disease	[69,70]
	Uteroplacental insufficiency	Rats	Insulin resistance, dyslipidemia, kidney disease, and hypertension	[71,72,73,74,75]
	Maternal hypoxia	Rats/mice/sheep	Obesity, hypertension, kidney disease, and CVD	[76,77,78,79,80]
	Maternal hypoprolactinemia	Rats	Obesity, insulin resistance, and kidney disease	[81,82]
	Maternal inflammation	Rats/mice	Obesity, metabolic disease, and hypertension	[83,84,85]
Drug use	Prenatal glucocorticoid exposure	Rats/mice/sheep	Obesity, insulin resistance, hypertension, and kidney disease	[86,87,88,89,90,91]
	Nicotine exposure	Rats	Hyperlipidemia, steatosis, hypertension, and kidney disease	[92,93,94,95]
	Ethanol exposure	Rats	Insulin resistance, kidney disease, and CVD	[96,97,98]
Chemical exposure	TCDD exposure	Rats/mice	Obesity, hypertension, kidney disease, and CVD	[99,100,101]
	DEHP exposure	Rats/mice	Obesity, insulin resistance, kidney disease, and hypertension	[102,103,104,105,106]
	BPA exposure	Rats	Obesity, dyslipidemia, insulin resistance, and hypertension	[107,108,109]

## Data Availability

The data are contained within the article.
